# Temporal disturbance of a model stream ecosystem by high microbial diversity from treated wastewater

**DOI:** 10.1002/mbo3.1347

**Published:** 2023-03-14

**Authors:** Tom L. Stach, Guido Sieber, Manan Shah, Sophie A. Simon, André Soares, Till L. V. Bornemann, Julia Plewka, Julian Künkel, Christian Becker, Folker Meyer, Jens Boenigk, Alexander J. Probst

**Affiliations:** ^1^ Environmental Metagenomics, Research Center One Health Ruhr of the University Alliance Ruhr, Faculty of Chemistry University of Duisburg‐Essen Essen Germany; ^2^ Department of Biodiversity University of Duisburg‐Essen Essen Germany; ^3^ Cologne Center for Genomics (CCG) Cologne Germany; ^4^ Institute for Artificial Intelligence University of Duisburg‐Essen Essen Germany; ^5^ Centre of Water and Environmental Research (ZWU) University of Duisburg‐Essen Essen Germany

**Keywords:** ARG, freshwater, mesocosm, metagenomics, microbiome, stressor

## Abstract

Microbial communities in freshwater streams play an essential role in ecosystem functioning via biogeochemical cycling. Yet, the impacts of treated wastewater influx into stream ecosystems on microbial strain diversity remain mostly unexplored. Here, we coupled full‐length 16S ribosomal RNA gene Nanopore sequencing and strain‐resolved metagenomics to investigate the impact of treated wastewater on a mesocosm system (AquaFlow) run with restored river water. Over 10 days, community Bray–Curtis dissimilarities between treated and control mesocosm decreased (0.57 ± 0.058 to 0.26 ± 0.046) based on *ribosomal protein S3* gene clustering, finally converging to nearly identical communities. Similarly, strain‐resolved metagenomics revealed a high diversity of bacteria and viruses after the introduction of treated wastewater; these microbes also decreased over time resulting in the same strain clusters in control and treatment at the end of the experiment. Specifically, 39.2% of viral strains detected in all samples were present after the introduction of treated wastewater only. Although bacteria present at low abundance in the treated wastewater introduced additional antibiotic resistance genes, signals of naturally occurring ARG‐encoding organisms resembled the resistome at the endpoint. Our results suggest that the previously stressed freshwater stream and its microbial community are resilient to a substantial introduction of treated wastewater.

## Graphical abstract

Wastewater represents a substantial portion of river microbiomes worldwide, yet its effect on stream microbiomes remains challenging to study. Here, we use a mesocosm setup combined with strain‐resolved metagenomics to unravel the impacts of treated wastewater on previously stressed stream water. The disturbance resulted in a significant shift of the microbiome, which did not persist over 10 days, suggesting that previously stressed stream microbiomes are resilient to the introduction of treated wastewater.

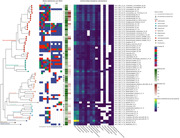

## 1 INTRODUCTION

Freshwater stream ecosystems are characterized by high biodiversity. Micro‐ and macroorganisms in these environments are responsible for essential ecosystem services with a major impact on biogeochemical cycling (Cardinale et al., 2012; Maavara et al., 2020; Ripl, 2003). These ecosystems have been exposed to environmental fluctuations on a daily, seasonal, and interannual timescale for centuries (Bucci et al., 2014; Burns et al., 1998; Hamid et al., 2020; Portillo et al., 2012). Over time, organisms have adapted to these natural disturbances (Crump et al., 2009; Lytle & Poff, 2004). Especially starting from the 20th century, additional anthropogenic stressors have been introduced to stream ecosystems such as water pollution, flow modification, habitat degradation, and climate change‐derived effects (Beattie et al., 2020; Dudgeon et al., 2006; Grill et al., 2019; Reid et al., 2019). One major anthropogenic stressor in stream ecosystems, particularly in urban areas, is the inflow of treated wastewater from wastewater treatment plants. The percentage of treated wastewater in the water body of an urban stream can reach from up to 30% during normal conditions to over 50% during low‐flow conditions, for example, during dry seasons (Drewes et al., 2018).

Although microbial activity has been considered important for ecosystem integrity (Cotner & Biddanda, 2002; Fasching et al., 2020), most studies have not considered microbial communities when studying the biome of streams (Gautam et al., 2022; Zeglin, 2015). Typically, experiments on stressor effects in streams have focused solely on higher organisms such as fish or invertebrates (Kim et al., 2020; Wright & Ryan, 2016), thus neglecting a major part of biodiversity, that is, prokaryotes and viruses. The latter are even considered to be a “blank spot on the map” in stream research (Peduzzi, 2016). Despite exceeding other microbes in diversity and particle number, information on viral interaction with organisms for example is missing so far (Bar‐On et al., 2018; Whitman et al., 1998). Thus, how microbial and viral communities are influenced by treated wastewater are a major gap of knowledge, which is nevertheless of great importance as river water serves as a major source of potable water in densely populated regions (Strathmann et al., 2016).

The AquaFlow mesocosm setup has previously been used to study the effects of flooding events on microeukaryotic communities (Graupner et al., 2017). In this study, the AquaFlow system was used to simulate an inflow of treated wastewater to understand its effect on microbial communities in river water. We studied prokaryotic and viral communities in the water phase for 10 consecutive days and in 6 mesocosms in parallel (three control systems and three treatment systems). The reference water used in our study was sourced from the near‐natural stream Boye, which had been used as an open sewer from the beginning of the last century until 2017. After a series of renaturation procedures, it has been fully restored since 2021 (Prati et al., 2022; Winking et al., 2014, 2016). In this study, we used a combination of full‐length 16S ribosomal RNA (rRNA) genes sequenced via Oxford Nanopore Technology (ONT) and genome‐resolved metagenomics on an Illumina NovaSeq. 6000 platform to conduct an in‐depth analysis of the prokaryotic community, ranging from community changes over time to strain‐resolved bacterial and viral analyses. Our results show that treated wastewater introduces a new microbial community to the existing stream ecosystem. This community differed not only at the bacterial phyla level but also strain level of the near‐identical bacterial and viral metagenome‐assembled genomes (MAGs) of cooccurring phyla. During an incubation period of 10 days in the AquaFlow systems, both treated and control experiments developed highly similar microbial communities after 10 days, suggesting the resilience of the river water community used herein regarding a pulse disturbance by treated wastewater.

## 2 MATERIALS AND METHODS

### 2.1 Study design and mesocosm setup

**Figure A1:**
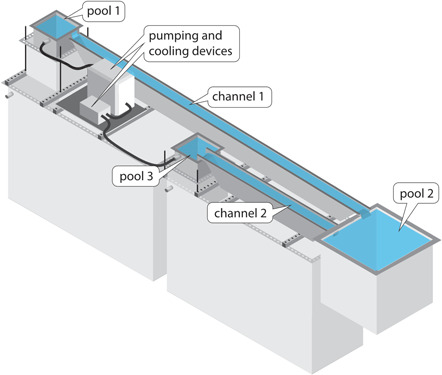
Schematic representation of the AquaFlow mesocosm setup as used in the presented study. One mesocosm system included three water tanks (~40, ~40, and ~270 L) connected by two steel channels (10 cm width and 4 and 2 m long) and a pump (10 L/min) to maintain a circular flow.

The AquaFlow mesocosm system was used to investigate the effects of treated wastewater on a natural stream ecosystem. The individual flow mesocosm systems are described in detail by Graupner et al. (2017). In short, one mesocosm system included three water tanks (~40, ~40, and ~270 L) connected by two steel channels (10 cm width and 4 and 2 m long) and a pump (10 L/min) to maintain a circular flow (shown as a scheme in Figure A1). The channels were filled with 60 L sediment taken from the river Boye (Germany; 51°33′19.7″ N and 6°56′38.3″ E, used as an open sewer until 2017 and fully restored in 2021) and homogenized in a concrete mixer before filling in. Per system, 350 L stream water from the same location as the sediment was prefiltered (200 μm pore size) and filled into the system. Throughout the experiment, the water temperature was kept at 19°C and natural sunlight was used as a light source. All six systems, three controls, and three treatments were run in parallel in February and March 2021.

Before the start of the experiment, an acclimatization phase of 14 days was performed, that is, running the systems as described and interconnecting the mesocosms each day until approximately 700 L were exchanged. Afterward, treated wastewater from the municipal wastewater treatment plant Schwerte (North Rhine‐Westphalia, Germany) was filled into three systems to reach a percentage of 33% treated wastewater (~120 L) per mesocosm after removing the same volume of control water beforehand in treatment systems. The wastewater treatment plant (WWTP) operated as explained in Rothe et al. (2021) with the following measurements at the effluent on the day of sampling: turbidity 3.4 NTU; chemical oxygen demand 16 mg/L; total phosphorus 0.23 mg/L; temperature 9.1°C; pH 6.81; and conductivity 928 µS/cm.

Thus, three systems served as controls (“C”) and three systems as treatments (“T”). Water samples were taken after 1 h, 12 h, 24 h, 2 days, 4 days, 7 days, and 10 days (samplings S1–S7). For each sample, 0.4 L water was filtered onside on 0.2 μm polycarbonate filters (Nucleopore; Cytiva) in duplicates, air‐dried, submerged in 400 μl DNA/RNA shield (Zymo Research), frozen in liquid nitrogen, and stored at −80°C until DNA extraction. DNA extraction was done at room temperature using the Zymo Quick DNA/RNA microprep plus kit (Zymo Research). Two filters with DNA/RNA shield solution were transferred in a Zymo BashingBeads Lysis Tube and homogenized using a FastPrep (MP Biomedical) with five steps at 5.5 m/s for 30 s and a resting step on the ice for 1 min after each step. Bashing bead tubes were centrifuged for 30 s, the supernatant was transferred to a 1.5 mL tube, and mixed with 400 μL lysis buffer. After transferring the samples to a Zymo‐Spin IC‐XM column, steps were performed according to the protocol until the addition of 30 μL RNAse/DNAse free water, incubation for 5 min, and centrifugation to elute the DNA. The latter was stored at −20°C until further processing.

### 2.2 DNA amplicon sequencing and processing

Full‐length 16S rRNA sequencing was performed for all 42 samples (systems C1–C3 and T1–T3; samplings S1–S7 each) using the 16S Barcoding kit (SQK‐RAB204; version RAB_9053_v1_revM_14Aug2019; Oxford Nanopore). The protocol was followed at each step if not mentioned otherwise using the LongAmp Taq 2× Master Mix (NEB) for PCR setup and Agentcourt AMPure XP beads (Beckman Coulter) for clean‐up. Instead of 10 ng genomic DNA, 15 ng was used as an input for the PCR. Sequencing was done on a MinION Mk1B using an FLO‐MIN106D flow cell, controlled by MinKNOW (v21.02.1). Per sample, at least 100,000 reads were sequenced. Raw sequencing signals were base‐called and demultiplexed using guppy (v5.0.7; dna_r9.4.1_450bps_hac.cfg).

Basecalled and demultiplexed 16S rRNA gene sequences were processed using the NanoCLUST pipeline (v1.0dev; UMAP settings ‐‐cluster_sel_epsilon 0.5 –umap_set_size 100000) (Rodríguez‐Pérez et al., 2021) based on the NCBI 16S rRNA database (v28.04.2021). Statistical analysis of operational taxonomic unit counts was performed using the R script MC_Stats (v1.2) as described in Weinmaier et al. (2015). Rarefaction and calculation of the Bray–Curtis dissimilarities were iterated 100 times and the average distance was calculated. Based on these dissimilarities principal coordinate analysis, nonmetric multidimensional scaling (NMDS), hierarchical clustering, rarefaction curve, diversity index, and multiple response permutation procedure (MRPP) were calculated using the R vegan package (Oksanen et al., 2013).

### 2.3 Metagenomic DNA sequencing, assembly, and annotation

Samples from the first and last sampling points (S1 and S7) were sent for metagenomic sequencing to the West German Genome Center (Cologne, Germany). Sequencing was done according to the Illumina PCR‐Free Protocol for Thermal Cycler, Low Input with a sequencing depth of 20 Gbp (Gigabase pair) (150 bp paired‐end reads) on a NovaSeq. 6000 with an S4 FlowCell.

Metagenomic sequences were quality checked using BBduk (Bushnell; https://jgi.doe.gov/data-and-tools/bbtools/bb-tools-user-guide/) and Sickle (Joshi & Fass, 2011) (quality score ≥ 20 and minimum read length ≥ 20 bp). The microbial community coverage by short‐read metagenomic sequences was estimated using Nonpareil3 (v3.4.1) (Rodriguez‐R et al., 2018).

Metagenomic paired‐end reads were individually assembled using a combined approach of metaviralSPAdes (Antipov et al., 2020) and metaSPAdes (Nurk et al., 2017) (SPAdes version 3.14.0). In short, reads were first assembled using metaviralSPAdes, generating scaffolds of viral origin. All metagenomic reads mapping (using Bowtie2; Langmead & Salzberg, 2012) to these viral scaffolds were excluded from the following assembly with metaSPAdes. Finally, both metaviralSPAdes and metaSPAdes assemblies were combined. Prediction of open reading frames was done for scaffolds equal or larger than 1 kbp using Prodigal (Hyatt et al., 2010) in meta mode and annotated using DIAMOND blast (Buchfink et al., 2015) (DIAMOND version 0.9.9; blastp ‐e 0.00001 ‐k 1) against an in‐house database called FunTaxDB based on UniRef100 (state February 2021; Bornemann et al., 2023; Suzek et al., 2007). The taxonomy of each scaffold was assigned as described in Bornemann et al. (2022). In brief, the scaffold taxonomy was assigned based on all proteins detected on the scaffold and the lowest taxonomic rank when more than 50% of the protein taxonomies agree. To calculate the average scaffold coverage, quality‐checked reads were mapped to scaffolds using Bowtie2 (Langmead & Salzberg, 2012). For all scaffolds, length, and GC content were calculated.

### 2.4 Comparison of taxonomic classification based on amplicon sequencing and metagenomics

Three approaches were compared to retrieve abundances of prokaryotes in our samples, that is, 16S rRNA gene data from ONT sequencing (described above), blasting of metagenomic reads to 16S rRNA genes from ONT, and *rpS3* (ribosomal protein S3) gene analysis from metagenomic data. The *rpS3* gene is a single‐copy gene containing both conserved and discriminatory regions and has been used for prokaryotic community analysis in metagenomic studies (Finstad et al., 2017; Smith & Wrighton, 2019; Zhong et al., 2022).

For blasting the quality‐checked short metagenomic reads to the results from 16S rRNA gene sequencing, the consensus sequence per cluster from NanoCLUST was taken as a basis. Using USEARCH, the short reads were blasted against the reference sequences (*e* value: 0.00001 and ‐top_hits_only), and all hits with more than three mismatches were discarded. Then, the average number of hits per cluster was calculated and the NCBI TaxID was added based on the NanoCLUST output.

*RpS3* genes were identified using a phylosift HMM (Hidden Markov Model) set (DNGNGWU00028; date: January 20, 2022) (Darling et al., 2014) in combination with hmmsearch (v3.2) at an *e* value cutoff of 1E−28. Additionally, annotation results using UniRef100 (described above) were searched for *rpS3* genes, and the respective taxonomy was attached. For statistical analyses of *rpS3* genes, representative genes were determined by clustering using USEARCH (‐cluster_fast ‐id 0.99). If the centroid of the cluster could be extended by 1 kb in both directions the sequence was chosen as a representative for the cluster. Otherwise, a noncentroid longest sequence that could be extended or just the longest sequence was selected. Then, quality‐passed reads were mapped to those representative *rpS3* gene sequences. To compare all three approaches, the TaxID and TaxString of the same NCBI database (date: January 2020) was used for taxonomic classification.

### 2.5 Screening for antibiotic resistance genes (ARGs)

Detection of ARGs was done as described by Rahman et al. (2018). In brief, ARGs were annotated to predicted genes using the Resfams HMM database (Gibson et al., 2015) using hmmsearch (v3.3) with the *‐‐cut_ga* flag. The latter was used to apply manually optimized profile‐specific gathering thresholds for reporting and inclusion of results (Gibson et al., 2015). Based on the Resfam output and scaffold coverage, a sample resistance gene summary was normalized to the number of sequenced reads. The Resfam metadata was used to translate the Resfam IDs to resistance mechanisms. Taxonomic annotation of scaffolds encoding for ARGs based on FunTaxDB (described above) was extracted to infer the underlying community harboring the resistome. For analysis of ARGs in MAGs, scaffolds encoding for resistances were matched to binned scaffolds, and hits of respective resistance mechanisms per MAGs were calculated.

### 2.6 Virus detection and analysis

Viral scaffolds were identified using VirSorter2 (v2.2.3; ‐‐high‐confidence‐only; Guo et al., 2021), deepvirfinder (v1.0.0; ‐l 1000) (Ren et al., 2020), and VIBRANT (v1.2.1) (Kieft et al., 2020). All hits were collected and checked using checkV (v0.7.0) (Nayfach et al., 2021). Only those viral scaffolds with a completeness higher than 25% were used for further analyses. All steps from raw reads until the detection of viral sequences were run in a Snakemake pipeline (Mölder et al., 2021).

### 2.7 Binning of assembled metagenomes and analysis of MAGs

Scaffolds equal or larger than 1 kb were used to generate MAGs in all 12 metagenomic samples. In short, ABAWACA (Brown et al., 2016), MaxBin2 (Wu et al., 2016), and MetaBAT2 (Kang et al., 2019) were run on all samples and an optimized set of bins was produced using DASTool (Sieber et al., 2018). For MaxBin2, both marker sets were used and a cross‐mapping over all samples using Bowtie2 was performed to use the differential coverage information as an input. The listed tools were wrapped in a Snakemake pipeline and run under Snakemake version 6.13.1 (Mölder et al., 2021).

All initial bins were manually curated using uBin (v0.9.14) (Bornemann et al., 2023) based on GC, coverage, taxonomy, and single‐copy core genes. After curation, all remaining non‐prophage viral sequences identified by checkV were removed from the MAGs as they likely represented contaminations. The resulting viral clean MAGs from all samples were dereplicated using dRep (v3.0.0) (Olm et al., 2017) using default settings and quality checked. In short, quality metrics were calculated using checkM (Parks et al., 2015) and Quast (Gurevich et al., 2013), transfer RNA (tRNA) genes were detected using tRNAscan‐SE (v2.0.7) (Chan et al., 2021), and rRNA genes were identified using RNAmmer (v1.2) (Lagesen et al., 2007). All results were combined and the quality of the MAG was assessed according to MIMAG standards by Bowers et al. (2017).

A standardized taxonomy assignment was done using GTDB‐Tk (v2.0.0, database release R207) (Chaumeil et al., 2020). To retrieve the taxonomy, the “classify_wf” pipeline was run, and an unrooted tree was created using the “infer” workflow.

### 2.8 Strain analysis of MAGs and viral genomes

Quality‐checked reads of all samples were mapped to the set of dereplicated MAGs using Bowtie2 and genes of MAGs were predicted using prodigal in normal mode. The inStrain (v1.5.5) (Olm et al., 2021) “profile” was run for each sample using the same set of dereplicated genomes followed by “inStrain compare” in database mode.

Viral genomes equal or larger than 3 kb and with minimal completeness of 25% were clustered using VIRIDIC (Moraru et al., 2020) with default parameters. From the resulting clusters, the centroid sequence was determined using USEARCH (v10.0.240) at an identity of 0.99. If more than one sequence remained per cluster, the longest sequence was selected for downstream analysis. These representative virus genomes were prepared as described before to run inStrain “profile” and “compare” over all samples.

### 2.9 Data visualization and significance testing

Final data polishing and visualization were done in R (R Core Team, 2022) using the packages ggplot2 (Wickham, 2016), tidyverse (Wickham et al., 2019), data.table (Dowle & Srinivasan, 2021), ggtree (Yu, 2020), ape (Paradis & Schliep, 2019), ggtreeExtra (Xu et al., 2021), nonpareil (Rodriguez‐R et al., 2018), and UpSetR (Gehlenborg, 2019). Paired *t* tests were performed to test for significant differences between 16S rRNA and *rpS3* gene analyses and AMR hits using the R package stats (R Core Team, 2022).

## 3 RESULTS

### 3.1 Wastewater‐induced shift in microbial community as revealed by full‐length 16S rRNA and rpS3 gene analysis

**Figure 1:**
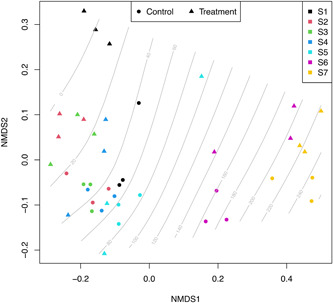
Nonmetric multidimensional scaling (NMDS) of full‐length 16S ribosomal RNA (rRNA) gene sequencing with curve fitting on incubation time in hours revealing a separation dependent on time and type. Sampling points refer to the time after the introduction of wastewater (S1–S7: 1 h, 12 h, 24 h, 2 days, 4 days, 7 days, and 10 days).

To determine the effect of wastewater on the microbial communities, we investigated 42 samples, that is, three controls and three treated samples each across seven time points, via 16S rRNA Nanopore sequencing. NMDS (Figure 1) revealed a change in the bacterial community with time along NMDS axis 1 and a separation of control and treatment along NDMS axis 2, indicating that time has a greater effect on the community than the addition of wastewater. This was confirmed via MRPP comparing the two sets of samples with a chance corrected within‐group agreement of 0.0675 for the addition of wastewater and 0.3775 for time, respectively (*p* = 0.001 for both tests).

**Figure A2:**
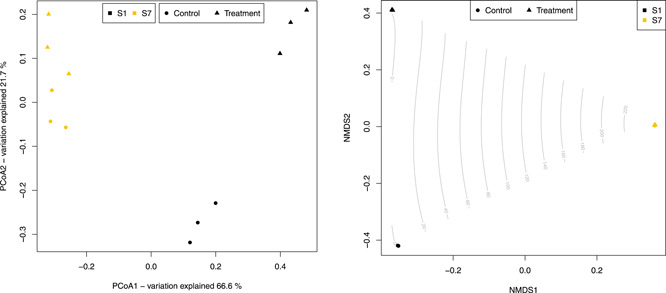
Principal coordinate analysis (PCoA) (left) and nonmetric multidimensional scaling (NMDS) (right) based on clustering of *rpS3* genes detected on metagenomic scaffolds. Samples are separated by treatment and time.

Based on these results the first and last sampling time points (12 samples in total, three for each time point with two time points for treatment and control, respectively) were selected for an in‐depth comparison via metagenomics. When comparing the 16S rRNA gene analysis with the *rpS3* gene analysis of these nine samples, we observed a similar pattern in ordination analyses (Figure A2).

**Figure 2:**
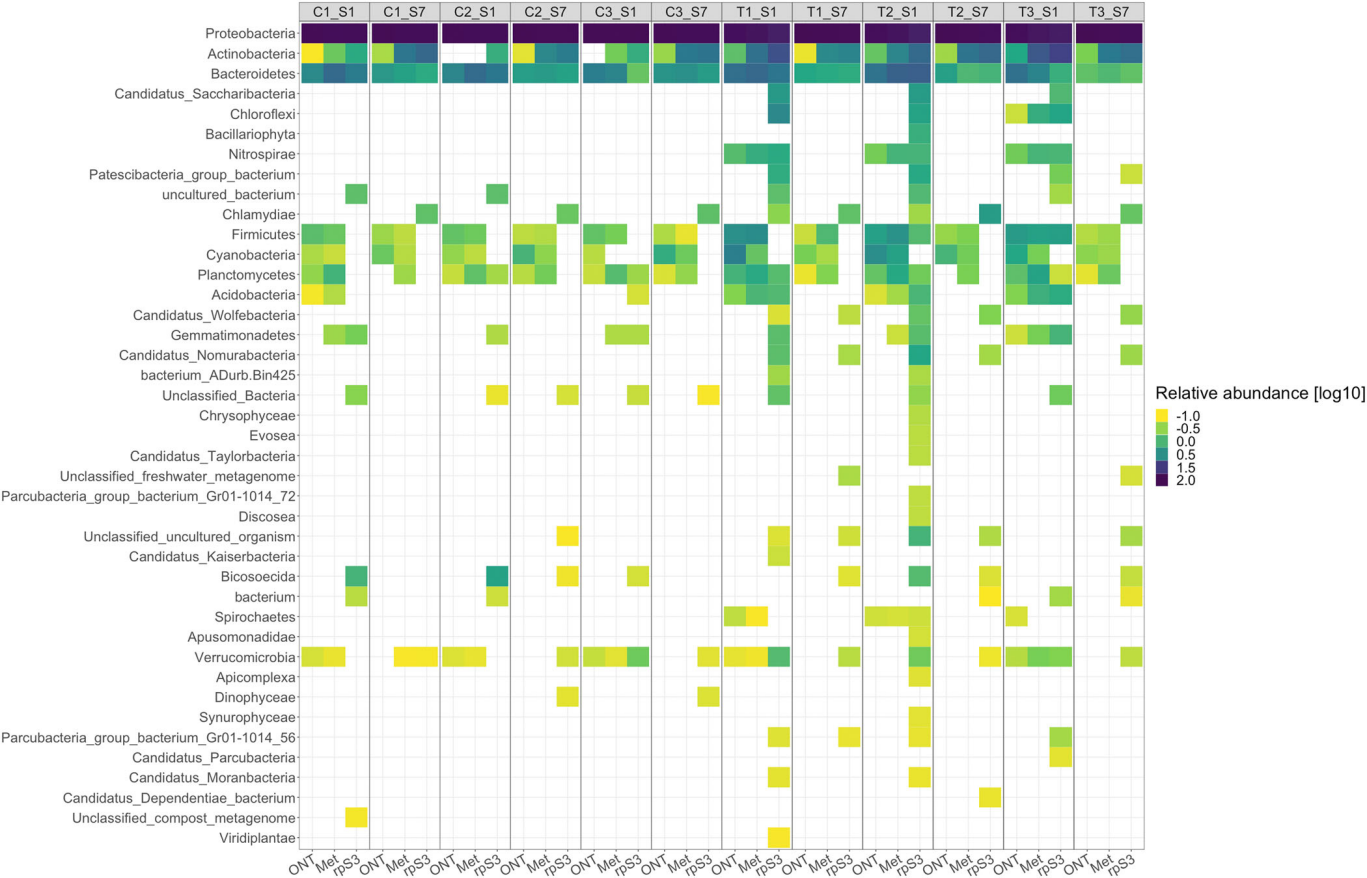
Phylum level relative abundance (≥0.1% abundance) of start and end point samples (“S1” and “S7”) of control (“C”) and treatment (“T”) systems based on three methods, that is, whole‐length 16S rRNA gene sequencing (“ONT”), blasting of metagenomic reads against reference sequences obtained by whole‐length 16S rRNA gene sequencing (“Met”), and rpS3 gene analysis based on metagenomics (“rpS3”).

Although beta‐diversity analyses revealed a similar pattern when using 16S rRNA and *rpS3* gene, 16S rRNA Nanopore sequencing results were further compared to the diversity detected via metagenomics using two independent approaches. First, we used USEARCH to assign short reads to the 16S rRNA genes from Nanopore sequencing to determine their relative abundance in the metagenomes. This approach enabled us to elucidate the amplification bias during 16S rRNA gene PCR. Yet, the bias in 16S rRNA gene analysis resulting from the possibility of multiple copies of the gene per genome is not reduced. Therefore, we directly used the relative abundances of *rpS3* genes, present as single copies in prokaryotic genomes, in assembled metagenomes and compared them to 16S rRNA gene data at the taxonomic level to determine the effect of primer bias combined with amplification bias in the analyses (Figure 2). Proteobacteria was the dominant phylum in relative abundance ranging from 59.04% based on *rpS3* genes analysis, to an average of 86.9% in abundance‐corrected 16S rRNA gene analysis up to 98.20% when using 16S rRNA genes from Nanopore only. Correction of relative abundance of Proteobacteria in 16S rRNA gene analysis using short reads revealed a lower abundance compared to Nanopore sequencing (*p* < 0.0001; paired *t* test), indicating an amplification bias favoring Proteobacteria. For all samples except C1_S1 and C3_S1, the detected relative abundance of Proteobacteria was lowest for *rpS3* gene analysis, highlighting a combinatorial effect of primer bias and PCR bias (*p* < 0.01 compared to 16S rRNA gene and *p* < 0.05 compared to abundance‐corrected 16S rRNA gene; both paired *t* test). Particularly the primer bias in 16S rRNA gene analysis was supported by the fact that *rpS3*‐based diversity analysis retrieved 44.4% more phyla (relative abundance ≥ 0.1%) on average over all 12 samples compared to 16S rRNA gene diversity, especially in the case of samples taken at the start of the experiment (56.3%).

### 3.2 Control and treatment develop the same strain clusters over time

Genome‐resolved metagenomics was used to investigate the community structure on a deeper taxonomic level. Overall samples, 376 manually curated MAGs were recovered and were dereplicated resulting in 100 reference MAGs (completeness ≥ 75%, contamination ≤ 25%) (Supporting Information: 2). Sixty‐six MAGs fulfilled the MIMAG criteria (Bowers et al., 2017) to be at least medium quality MAGs (e.g., selecting MAGs with contamination ≤ 10%) and were present in at least two samples according to inStrain and thus comparable at the strain level.

**Figure 3:**
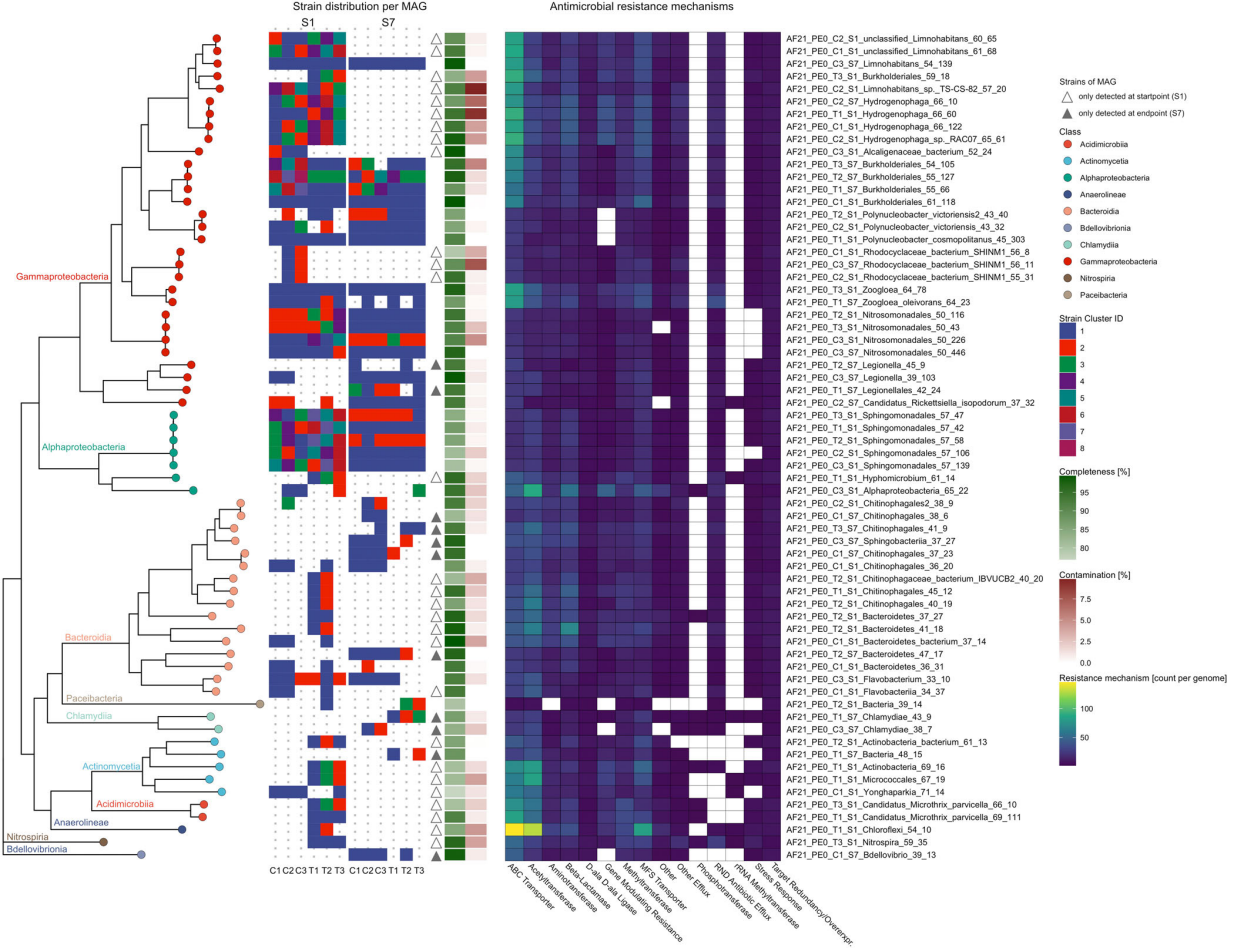
Distribution of metagenome‐assembled genome (MAG) strain clusters in metagenomic samples faceted by start and endpoint sampling. Strain cluster IDs are referring to the respective MAG only. For example, if the same strain of a certain MAG is present in all samples, they are shown as strain cluster one and if a sample contains another strain of the same MAG, this sample is color coded as strain cluster two, and so on. A genome is considered present if at least 50% of the genome is covered (breadth = 0.5). Only MAGs fulfilling MIMAG requirements for medium‐quality MAGs present in at least two samples are considered (Bowers et al., 2017). MAGs that were only detected at the start and endpoint sampling were labeled with empty and filled triangles, respectively. Antimicrobial resistance mechanisms were assigned to the MAGs based on Resfams HMMs.

Similar to marker gene analyses (see above), Proteobacteria represented the highest occurring phylum also among MAGs (Figure 3). Of the 66 MAGs in total, 28 were only detected in the initial sampling and 11 only in endpoint sampling (labeled with empty and filled triangles, respectively). Seventeen MAGs were exclusively detected in treatment samples and seven only in control samples. The number of strain clusters per MAG reduced from 1.8 ± 0.90 (*n* = 40 MAG clusters) to 1.29 ± 0.63 (*n* = 34) for control and from 2.0 ± 0.91 (*n* = 49) to 1.27 ± 0.52 (*n* = 33) for treatment from S1 to S7. MAGs belonging to lower abundant classes (Figure 3) were mostly found in samples treated with wastewater, for example, Acidimicrobiia, Nitrospiria, or Bacteroidia.

**Figure 4:**
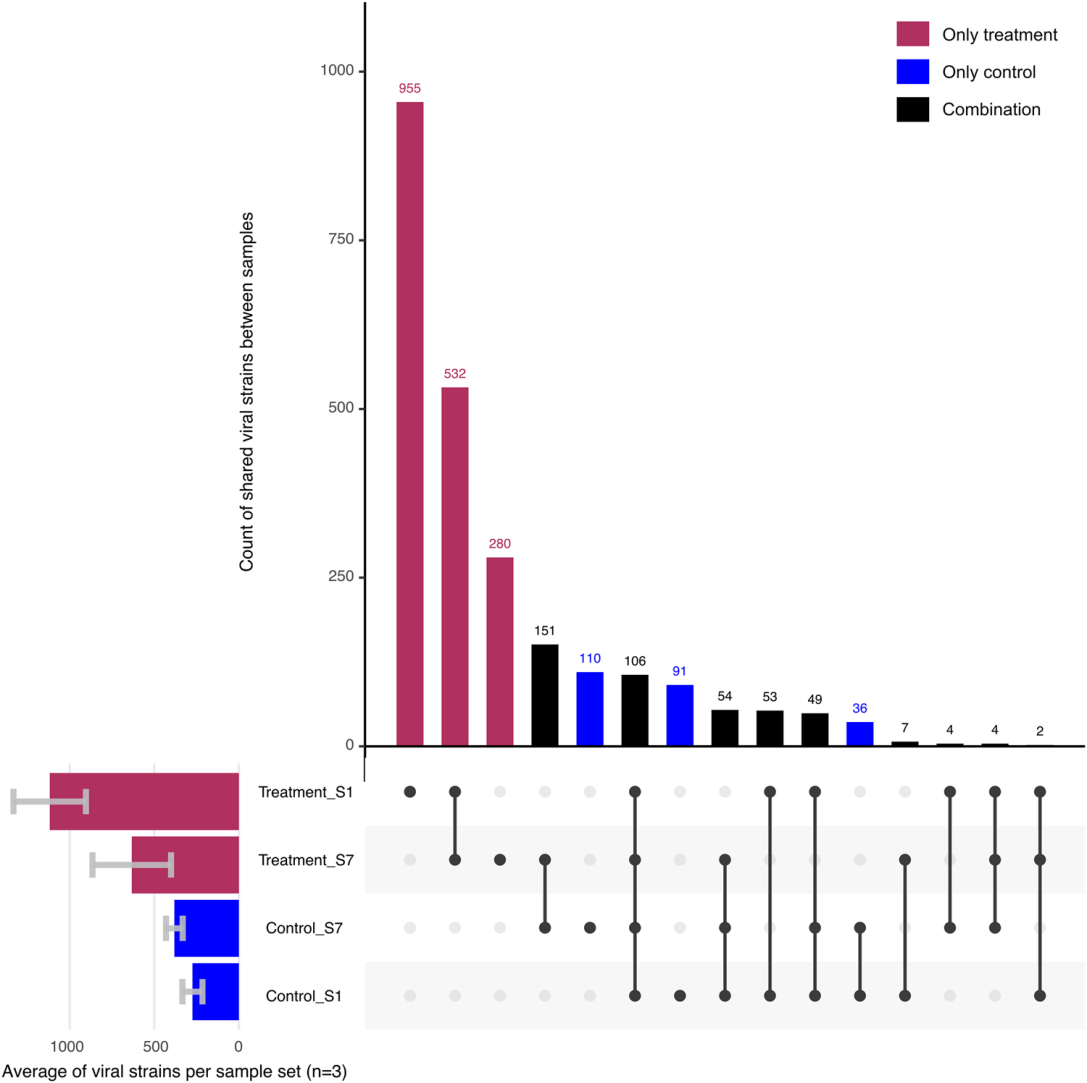
Counts of viral subclusters, that is, viral strains, present in treatment and control at start and endpoint sampling as detected by inStrain (module compare). Connected samples indicate the count of shared viral strains of respective samples.

This strain analysis of bacteria was also performed on quasi‐species of viruses detected in the metagenomes. Overall, 2336 representative viral scaffolds were identified, of which 1713 were present in at least two samples and used for further analyses. 128 viral scaffolds were detected in control samples only, whereas 1164 viral scaffolds were exclusively detected in samples treated with wastewater. InStrain detected 2434 subclusters of viral scaffolds, that is, viral strains, across different samples (Figure 4). In treatment samples, 1767 (72.6%) viral strains were present either at the start or end point or detected in both with the highest percentage at the start (955; 39.2%). In contrast to treatment samples, viral strains present in control samples only made up 9.7%, highlighting a high diversity of viral strains introduced via treated wastewater.

### 3.3 Stress response increase in control and treatment samples

**Figure 5:**
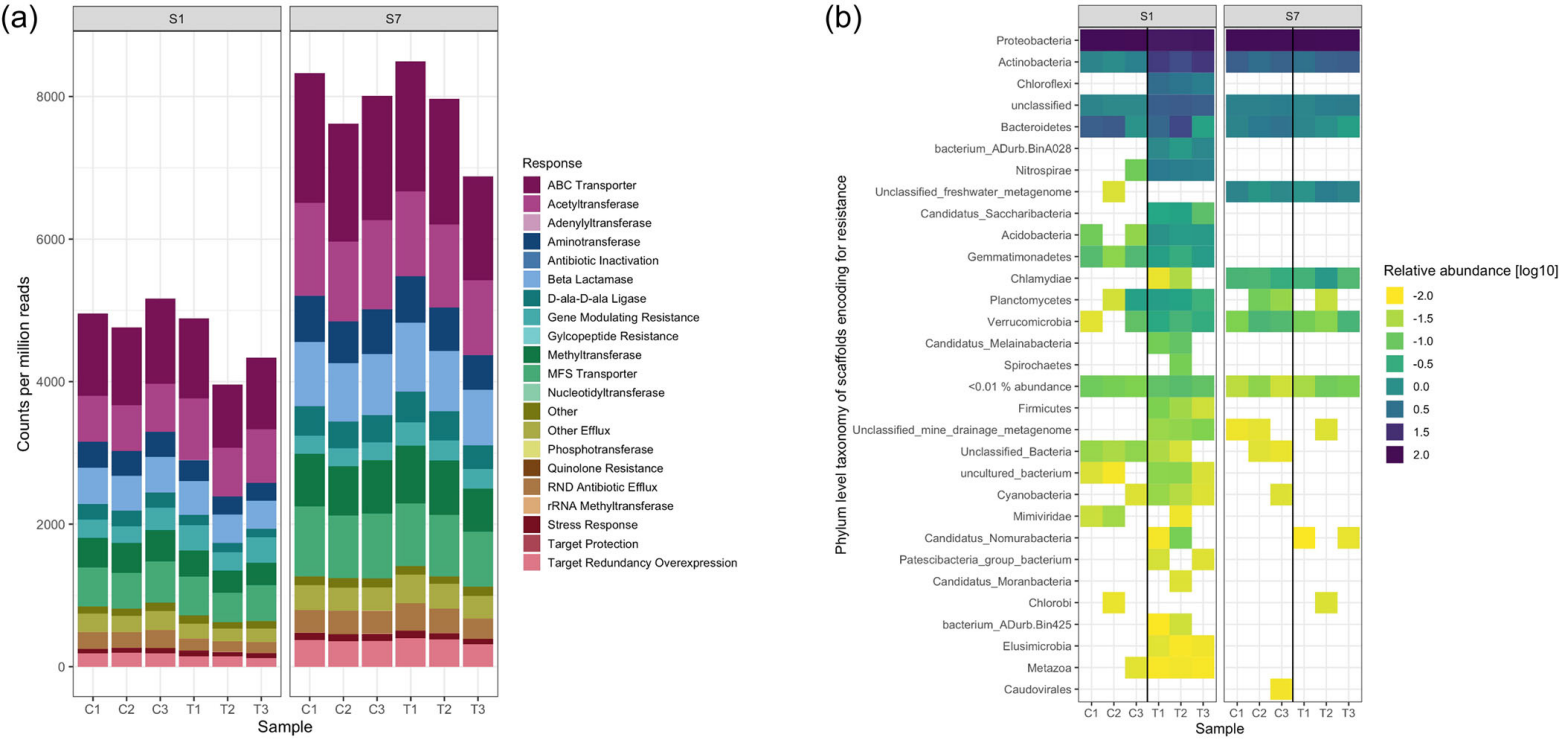
Stress response encoded on metagenomic scaffolds. Total counts (a) were normalized using sequencing depth and scaffold coverage. Scaffolds encoding for stress response were extracted and a phylum‐level taxonomy was annotated (b). A relative abundance of 0.5 would reflect that 50% of counts were detected on scaffolds assigned to that phylum.

Assembled metagenomes were scanned for antimicrobial resistance genes using the Resfams HMM database (Gibson et al., 2015), resulting in ATP‐binding cassette transporter (ABC transporter), acetyltransferase, major facilitator superfamily transporter (MFS Transporter), and β‐lactamases with the highest counts per million reads (Supporting Information: 1). Sums of counts were significantly higher for endpoint samples compared to the start of the experiment (*p* = 2.13 × 10^−14^; Figure 5a). The taxonomic profiles of scaffolds carrying these resistance genes were determined using the uBin package (Bornemann et al., 2023), and their distribution is shown in Figure 5b. In wastewater‐treated samples, 57.98%–61.74% of resistance gene hits were assigned to Proteobacteria at the initial sampling, in contrast to 90.20%–94.92% in control samples. The share of less abundant phyla encoding for resistance genes is reduced from S1 to S7 in treatment samples, resulting in the same distribution compared to the control samples. This is also supported by the ARG analysis in MAGs (Figure 3). Genomes encoding for resistances were introduced by treated wastewater contributing to a more diverse resistome at the beginning, for example, MAG AF21_PE0_T1_S1_Chloroflexi_54_10. At sampling point S7 however, mainly MAGs belonging to the Proteobacteria remain in both control and treatment, forming the resistome.

## 4 DISCUSSION

Stream ecosystems harbor a rich biodiversity, shape and connect ecosystems, and serve humankind in many ways (Dudgeon et al., 2006; Honey‐Rosés et al., 2013). Such ecosystem services are dependent on healthy microbial communities, which can be stressed by natural and anthropogenic influences (Lin et al., 2019). Effluents of WWTPs represent a ubiquitous stressor for freshwater streams, a general source of drinking water production. While the microbial community of WWTP effluents has already been investigated directly at the outlet (Newton et al., 2022) as well as in an upstream/downstream approach (Chaudhary et al., 2018), the temporal development of the microbial community for a longer period after addition of wastewater has not been investigated so far. Surveying such a disturbance in real stream systems is challenging due to the heterogeneous nature of fluvial ecosystems, for example, different flow conditions, influence from the shore (e.g., leaf litter) or changing weather conditions (Bastias et al., 2020; Beisel et al., 2000). Mesocosm systems like the AquaFlow system, however, enable such investigations with the trade‐off of studying a closed system (Graupner et al., 2017; Röhl et al., 2018). In this study, we replaced one‐third of the water in three AquaFlow systems with treated wastewater from a municipal WWTP and investigated the microbiome for 10 days using 16S rRNA gene and shotgun sequencing. The control system was only filled with water from the stream Boye, a previous open sewer that has been wastewater free since 2017 and restored since 2021.

**Figure A3:**
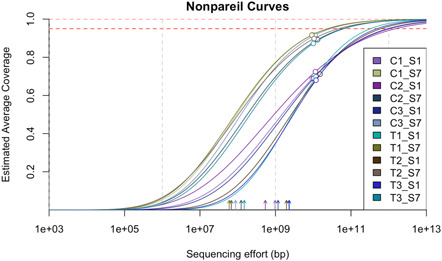
Nonpareil curves for metagenomic samples. Empty circles indicate the actual sequencing effort and arrows indicate the community diversity. Horizontal red dashed lines represent 95% and 99% coverage. All samples investigated had more than 60% coverage in diversity.

As expected, the introduction of treated wastewater resulted in a significant disturbance of the microbial community as revealed by both marker genes used in this study, with commonly known phyla introduced by treated wastewater (Newton et al., 2022). Yet, only *rpS3* gene analysis based on metagenomic sequencing was able to detect also lower abundant phyla, which we attribute to primer biases in amplicon analyses. Treated wastewater introduced not only new phyla but also new strains of MAGs and viruses, highlighting its impact on the microbial community composition. As presented, counts of both bacterial and viral clusters introduced by treated wastewater, but also those which were already present in the near‐natural stream, were reduced over the incubation period of ten days in the mesocosm system. This is also reflected by the decrease of Nonpareil diversity (Figure A3) from start to endpoint for all systems and the increase of estimated sequencing coverage at comparable sequencing depth (22.4 ± 3.5 Gbp), suggesting that the setup in combination with the factor time itself serves as a driver for the microbial community.

A major public concern about WWTP effluents released into streams is the introduction of antibiotic‐resistant bacteria and ARGs along with their propagation in natural ecosystems (Araújo et al., 2010; Ferreira da Silva et al., 2007; Sekizuka et al., 2022). Our results showed that the added treated wastewater introduced new phyla encoding for stress response systems including antimicrobial resistances. The total count of stress response genes did not increase compared to control systems, suggesting that the natural resistome was only changed to a more diverse resistome originating from the treated wastewater. After 10 days of incubation, the near‐natural stream microbiome was solely responsible for the composition of the resistome resulting in an increase of stress response genes. One explanation could be that the control stream water originated from a previously stressed ecosystem that was under influence of wastewater for centuries and only freed from wastewater 4 years before sampling and restored in the year of sampling. Thus, the restored stream microbiome should still be adapted to the environmental change that the mesocosm setup represents. However, the increase of stress response systems over time along with the factor time as the major driver of the microbial community in 16S rRNA gene analyses, suggests that the community in the AquaFlow system is under constant development. Nevertheless, the effect of stressors like wastewater introduction could be elucidated and the introduction of strains of bacteria and viruses and stress response systems could be detected.

Our results suggest a limited temporal disturbance of microbial communities by wastewater bacteria, viruses, and ARGs in restored ecosystems. Yet, the persistence of biological entities was short, arguably because fecal organisms, for example, have a limited survival time in open water columns (Brooks et al., 2015). It has also been shown previously that highly polluted urban rivers might have a higher resilience to such a pulse disturbance than more natural rivers (García‐Armisen et al., 2014). Our results suggest that despite comprehensive measures the river Boye is likely still in a stressed state and the microbial community is resilient to pulse disturbances of treated wastewater. Future studies including a pristine freshwater stream should be able to verify this hypothesis. From a public health perspective, the return to the equilibrium state of the control counters concerns about the impacts of WWTP effluents, however, it is unclear if the same holds true for rivers not previously stressed. In addition, the duration of the temporary disturbance might impact the microbiome resilience.

## 5 CONCLUSIONS

In conclusion, investigation of long‐term effects caused by anthropogenic disturbances in stream systems is currently only possible using mesocosm systems like the presented AquaFlow setup. Here, we show that the introduction of treated wastewater resulted in a drastic temporal disturbance of a restored freshwater stream microbiome by introducing bacterial phyla, viruses, and a more diverse resistome. Yet, the introduction showed no lasting effect on the microbial community, and both control and treatment developed to a similar endpoint community. This suggests that the microbiome of a previously stressed river might be resilient to a drastic, but temporary disturbance.

## AUTHOR CONTRIBUTIONS

**Tom L. Stach**: Data curation (lead); formal analysis (lead); investigation (equal); visualization (lead); writing—original draft (lead); writing—review and editing (equal). **Guido Sieber**: Conceptualization (equal); methodology (equal); writing—review and editing (supporting). **Manan Shah**: Conceptualization (equal); methodology (equal); writing—review and editing (supporting). **Sophie A. Simon**: Methodology (equal); writing—review and editing (supporting). **André Soares**: Methodology (supporting); writing—review and editing (supporting). **Till L. V. Bornemann**: Methodology (supporting); writing—review and editing (supporting). **Julia Plewka**: Methodology (equal); writing—review and editing (supporting). **Julian Künkel**: Investigation (supporting); writing—review and editing (supporting). **Christian Becker**: Resources (supporting). **Folker Meyer**: Writing—review and editing (supporting). **Jens Boenigk**: Conceptualization (equal); funding acquisition (lead); resources (equal); supervision (equal); writing—review and editing (supporting). **Alexander J. Probst**: Conceptualization (lead); funding acquisition (lead); resources (equal); supervision (equal); writing— original draft (lead); writing—review and editing (equal).

## CONFLICT OF INTEREST STATEMENT

None declared.

## ETHICS STATEMENT

None required.

## Supporting information

Supporting information.

Supporting information.

Supporting information.

Supporting information.

## Figures and Tables

**Figure A4:**
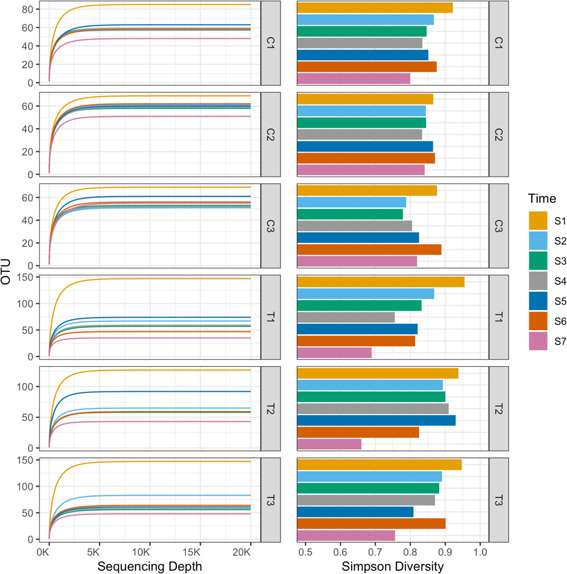
Rarefaction curve (left) and Simpson diversity (right) based on 16S ribosomal RNA (rRNA) gene analysis. Especially for treatment samples, diversity was highest at the start and reduced towards the end of the experiment. OTU, operational taxonomic unit.

**Figure A5:**
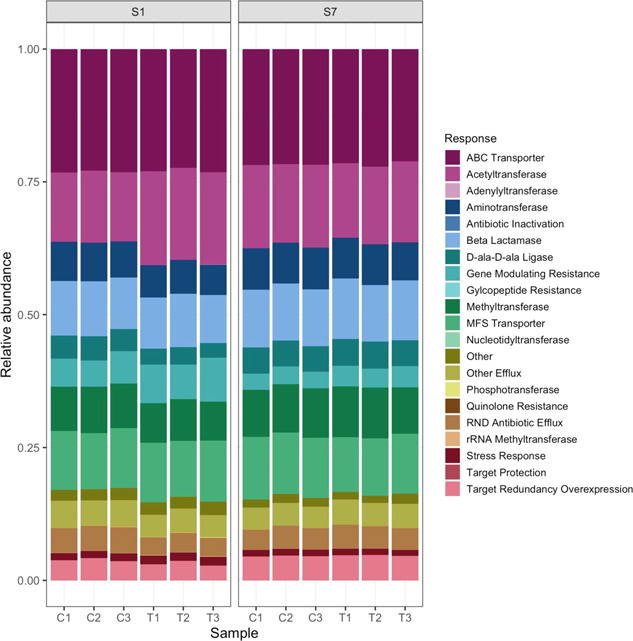
Stress response encoded on metagenomic scaffolds with relative abundances per mechanism and sample. ABC, ATP‐binding cassette; MFS, major facilitator superfamily; rRNA, ribosomal RNA; RND, resistance nodulation division.

## Data Availability

Raw sequencing data and MAGs have been deposited at SRA and GenBank, respectively, and are accessible under the BioProject PRJNA874517: https://www.ncbi.nlm.nih.gov/bioproject/PRJNA874517.

## 1 Scheme of an AquaFlow mesocosm system

Figure A1

## 2 Statistical analyses of start and end point samples based on rpS3 gene clustering

Figure A2

## 3 Coverage of shotgun metagenomic sequencing diversity

The diversity of start‐ and endpoint samples estimated by Nonpareil3 (Rodriguez‐R et al., 2018) ranged for initial treatment samples from Nonpareil diversity (Nd) 21.39 to 21.56 (*n* = 3) and for initial control samples from Nd 20.10 to 20.88 (*n* = 3). All endpoint samples ranged from Nd 17.89 to 18.82 (*n* = 6). The sequencing coverage was significantly higher for endpoint samples based on the redundancy estimation from Nonpareil (*n* = 12, *t* test p = 3.008 × 10^−8^) with an average of 0.69 ± 0.019 for start‐point and 0.86 ± 0.017 for end‐point samples. Corresponding Nonpareil curves are shown in Figure  A3.

## 4 Rarefaction curve and diversity of 16S rRNA gene analysis

Figure A4

## 5 Relative abundances of antimicrobial resistance mechanism

Figure A5
